# Demagnetization Severity Detection in Permanent Magnet Synchronous Motors Based on Temperature Signal and Convolutional Neural Network

**DOI:** 10.3390/s26092631

**Published:** 2026-04-24

**Authors:** Zhiqiang Wang, Shihao Yan, Haodong Sun, Xin Gu, Zhichen Lin, Kefei Zhu

**Affiliations:** 1 School of Electrical Engineering, Tiangong University, Tianjin 300387, China; 2331070875@tiangong.edu.cn (S.Y.); 2431070887@tiangong.edu.cn (H.S.); guxin@tiangong.edu.cn (X.G.); 2 Zhejiang University Advanced Electrical Equipment Innovation Center, Hangzhou 311107, China; 3 Shanghai Electric Drive Co., Ltd., Shanghai 200240, China; 4 School of Mechatronic Engineering and Automation, Shanghai University, Shanghai 200444, China

**Keywords:** demagnetization severity detection, permanent magnet synchronous motor, local demagnetization, temperature, Convolutional Neural Network

## Abstract

To address the difficulty of detecting demagnetization severity in permanent magnet synchronous motors (PMSMs), this paper proposes a demagnetization severity detection method based on temperature signal and Convolutional Neural Network (CNN). First, the differences between local demagnetization and eccentricity fault in stator current harmonics are analyzed from an electromagnetic perspective, and fast Fourier transform (FFT) is used for frequency-domain analysis of the stator current to identify local demagnetization faults. On this basis, an electromagnetic–thermal coupling model is established by considering motor losses and heat dissipation boundary conditions to obtain the winding temperatures under different demagnetization severities and operating conditions. Furthermore, the temperature time series, together with speed and load torque, is constructed into a three-dimensional state space, and the proposed Conditionally Modulated Multi-Scale Convolutional Neural Network (CMSCNN) is introduced for feature learning to achieve demagnetization severity detection. Experimental results show that the proposed method achieves an average detection accuracy of 98.06% on the simulation test set and outperforms the baseline CNN model. On measured data collected from the faulty prototype, the average detection accuracy reaches 93.34%, verifying the effectiveness of the proposed method for demagnetization severity detection.

## 1. Introduction

Permanent Magnet Synchronous Motors (PMSMs) are widely used in industrial drive systems due to their advantages of high power density and high efficiency. However, during practical operation, the permanent magnets are susceptible to high temperature, strong armature demagnetizing fields, chemical corrosion, and mechanical impact, which may lead to magnetic property degradation and eventually induce demagnetization faults [1,2,3]. Demagnetization reduces the no-load back electromotive force (back-EMF). To maintain the required torque output, the stator current must be increased, thereby resulting in higher losses and temperature rise and further aggravating the demagnetization process [4,5,6]. As the operating frequency increases, the motor loss and temperature-rise issues become more pronounced [7]. Demagnetization faults can be mainly classified into uniform demagnetization and local demagnetization. Uniform demagnetization is generally caused by abnormal temperature rise or material aging, while the three-phase parameters of the motor remain symmetrical. In contrast, local demagnetization destroys the symmetry of the air-gap magnetic field, leading to air-gap flux density distortion, increased vibration and noise, and even permanent motor damage in severe cases [8,9,10,11]. Therefore, accurate diagnosis of local demagnetization faults is of great significance for improving the operational reliability of PMSMs. In addition, the performance of complex permanent magnet electromagnetic devices can also be investigated through structural design, optimization analysis, and experimental validation [12].

Demagnetization fault diagnosis should not only determine whether a fault has occurred, but also pay attention to its development stage and severity. Accurate detection of demagnetization severity helps ensure the safe operation of motors, reduce energy consumption, extend service life, and provide a basis for hierarchical management and refined decision-making for maintenance personnel [13,14,15]. For example, under slight demagnetization, the motor can continue to operate and maintenance can be reasonably scheduled; under moderate demagnetization, protective measures such as derated operation can be adopted; and under severe demagnetization, the motor should be shut down and inspected in a timely manner to prevent fault aggravation or system failure. Therefore, demagnetization severity detection is of great engineering significance for realizing hierarchical motor management, risk assessment, and full-life-cycle health monitoring [16,17,18]. Relevant studies have combined analytical modeling, finite element analysis, and experimental validation for the performance analysis of complex permanent magnet structures [19]. At present, PMSM demagnetization fault diagnosis methods can be mainly classified into three categories: feature signal-based methods, analytical model-based methods, and artificial intelligence-based methods [20,21,22].

Feature signal-based demagnetization fault diagnosis methods mainly rely on the electromagnetic and mechanical response quantities generated during motor operation. By extracting feature parameters related to demagnetization and constructing corresponding diagnostic indicators, fault diagnosis can be achieved. Reference [23] analyzed the influence of permanent magnet demagnetization on the air-gap flux density distribution and the harmonic characteristics of the stator-induced electromotive force, thereby providing a theoretical basis for demagnetization diagnosis. Reference [24] combined vibration signals and stator current signals to develop a deep learning model based on transfer learning pretraining, achieving demagnetization fault identification in PMSMs. Reference [25] realized demagnetization fault diagnosis of PMSMs by extracting stator tooth flux signals. Feature signal-based methods are relatively straightforward to implement and have a solid foundation for engineering applications; however, under complex operating conditions, they are easily affected by load fluctuations, noise, and harmonics.

Analytical model-based demagnetization fault diagnosis methods are developed on the basis of the electromagnetic and electromechanical coupling characteristics of motors. By establishing mathematical models or equivalent parameter models incorporating demagnetization effects, observable quantities can be constructed to achieve fault diagnosis. Reference [26] proposed a parameter identification method based on an analytical motor model. By calculating the inductance variation under demagnetization conditions and combining it with the least-squares algorithm, the flux linkage estimation accuracy was improved, thereby enabling the identification of demagnetization faults and their severity. Reference [27] proposed a permanent magnet flux linkage identification method based on speed harmonics. By establishing the relationship between flux linkage and speed harmonics, demagnetization fault detection of permanent magnets was achieved. Analytical model-based methods offer strong physical interpretability, but they generally rely heavily on model accuracy and parameter precision.

Artificial intelligence-based demagnetization fault diagnosis methods adopt a data-driven strategy to achieve fault identification and demagnetization severity detection by learning the relationship between fault signals and demagnetization. Reference [28] proposed a least-squares support vector machine method combining S-transform time–frequency feature extraction and particle swarm optimization, thereby realizing the joint identification of the demagnetized pole position and demagnetization severity. Reference [29] constructed a Convolutional Neural Network model using stator current data to achieve early detection of permanent magnet demagnetization faults. Reference [30] proposed a multi-source information fusion diagnosis method, in which stator current and torque signals were combined with a Convolutional Neural Network incorporating an attention mechanism to realize demagnetization fault identification. Artificial intelligence-based methods are well suited for fault detection under complex operating conditions, but their performance largely depends on sample quality, dataset size, and training conditions.

In practical operating environments, PMSMs often operate under complex conditions such as variable speed and variable load. Factors including load fluctuations, inverter switching harmonics, and electromagnetic noise may interfere with electromagnetic signals, causing the demagnetization-related features to couple with normal components and thereby reducing fault distinguishability. Meanwhile, inverter PWM harmonics can also increase the motor iron loss, thereby further affecting operating efficiency and temperature rise [31]. Traditional demagnetization detection methods mainly rely on manually constructed features or accurate electromagnetic models and are highly dependent on motor and control parameters. Therefore, under complex operating conditions, these methods are prone to reduced detection accuracy and even misjudgment [32,33,34].

In contrast, temperature signals can reflect the combined response of motor losses and magnetic property variations from a thermal perspective and exhibit strong anti-interference capability [35]. After permanent magnet demagnetization occurs, the air-gap flux density distribution and the electromagnetic energy conversion process are altered, which further leads to increased internal motor losses and affects the temperature rise of the stator windings. On this basis, this paper proposes a demagnetization severity detection method based on temperature signals and a Convolutional Neural Network. First, from the perspective of electromagnetic mechanisms, the differences between local demagnetization and eccentricity fault in the harmonic characteristics of stator current are analyzed, and fast Fourier transform (FFT) is employed to perform frequency-domain analysis of the stator current signal, thereby enabling demagnetization fault identification. On this basis, an electromagnetic–thermal coupling model is established to obtain the winding temperature time series under different demagnetization severities as well as different operating conditions of speed and load torque. Subsequently, temperature information, together with speed and load torque, is used as the input to construct a three-dimensional state space representing the operating characteristics of the motor, and the proposed Conditionally Modulated Multi-Scale Convolutional Neural Network (CMSCNN) is introduced for feature extraction to achieve demagnetization severity detection. This method uses the temperature signal as the primary information source and constructs a three-dimensional state space by incorporating rotational speed and load torque, thereby improving the stability of demagnetization severity detection under multiple operating conditions. Meanwhile, by introducing conditional modulation, multi-scale convolution, and a residual structure, the model ability to extract temperature features and the capability to identify different demagnetization severities are enhanced.

## 2. Analysis of Demagnetization Faults in Permanent Magnet Synchronous Motors

When local demagnetization occurs in a PMSM, the magnetic properties of the rotor permanent magnets deteriorate, thereby destroying the original symmetry of the air-gap flux density. Therefore, the variation law of the air-gap flux density under demagnetization conditions and its fault-harmonic response in the stator current are first analyzed from an electromagnetic perspective, so as to provide a theoretical basis for subsequent feature extraction of demagnetization faults.

### 2.1. Analysis of Local Demagnetization Faults

Under healthy operating conditions, the flux density established by the permanent magnets along the circumferential direction of the air gap exhibits a periodic distribution. The spatial flux density can be expressed as the superposition of the fundamental component and a number of higher-order harmonic components:

(1) $$B_{0}(\theta )=\sum_{m=1}^{\infty }B_{m}\cos(mp\theta )$$

where *p* denotes the number of pole pairs, *θ* is the mechanical angle, and *m* is the harmonic order.

Assume that demagnetization occurs in one magnetic pole of the motor. In this case, the amplitude of the induced electromotive force generated when this pole acts on the stator winding will decrease. To describe this effect, the sinusoidal back electromotive force *E_solt_* under healthy conditions is taken as the reference, and the potential attenuation caused by demagnetization is represented by subtracting a disturbance term *y*(*t*). Here, *y*(*t*) can be expressed as the product of a fundamental sinusoidal wave and a square wave *x*(*t*), and a demagnetization coefficient is introduced to characterize the attenuation level. The function *x*(*t*) is used to describe the time interval during which the demagnetized pole acts on the winding. Its frequency is *f_e_*/*p*, and its duty cycle is *d* = 1/2*p*. The Fourier series expansion of *x*(*t*) is given as follows:

(2) $$x(t)=\frac{1}{2p}+\sum_{n=1}^{\infty }\frac{2}{n\pi }\sin(\pi nd)\cos(\frac{2n\pi f_{e}t}{p})$$

where *f_e_* denotes the fundamental frequency of the motor. Then, *y*(*t*) can be expressed as follows:

(3) $$y(t)=\frac{k_{de}}{2p}\cos(2\pi f_{e}t)+k_{de}\sum_{n=1}^{\infty }\frac{1}{n\pi }\sin(\frac{\pi n}{2p})\cos(2\pi f_{e}t(1\pm \frac{n}{p}))$$

where *k_de_* denotes the demagnetization severity of a single permanent magnet. The back electromotive force *E_de_slot_* induced by the rotor of the faulty motor in one stator slot can be expressed as follows:

(4) $$E_{de\_slot}=V_{slot}(1-\frac{K_{de}}{2p})\cos(2\pi f_{e}t)-K_{de}V_{slot}\sum_{n=1}^{\infty }\frac{1}{n\pi }\sin(\frac{\pi n}{2p})\cos(2\pi f_{e}t(1\pm \frac{n}{p}))$$

where *V_slot_* denotes the amplitude of the back electromotive force induced in one stator slot under healthy operating conditions.

According to (4), demagnetization of a single permanent magnet leads to a reduction in the amplitude of the induced back electromotive force *E_de_slot_*. Theoretically, after the fault occurs, the amplitude decreases to (1 − *k_de_*/2*p*) times its original value. In addition, the demagnetization fault introduces fault-related harmonic components into the back electromotive force waveform. In AC permanent magnet synchronous motors, the voltage and current are periodic functions, and the back electromotive force is also a periodic function. Therefore, the fault components appearing in the back electromotive force will also give rise to corresponding specific fault components in the stator current. Their frequencies are given by:

(5) $$f_{ec}=f_{e}(1\pm \frac{n}{p})$$

### 2.2. Differential Analysis of Fault Characteristics

Air-gap magnetic field distortion is not unique to local demagnetization faults. Rotor eccentricity faults can also destroy the symmetry of the air-gap magnetic field, thereby generating additional harmonics in the stator current. If fault diagnosis is performed solely based on the presence or absence of harmonics, different faults may be easily confused. Therefore, it is necessary to further analyze the formation mechanism of air-gap magnetic field distortion under eccentricity fault conditions and its corresponding current harmonic characteristics, and then compare them with those of demagnetization faults, so as to improve the accuracy of demagnetization fault diagnosis.

In this paper, dynamic eccentricity fault is taken as the object of analysis. Under dynamic eccentricity conditions, the center of rotation of the rotor coincides with the geometric center of the stator, whereas the geometric center of the rotor itself is offset relative to that of the stator. The air-gap length *δ* after eccentricity can be expressed as follows:

(6) $$\delta (\varphi ,t)=\delta_{h}(1-\frac{e}{\delta_{h}}\cos(w_{r}t-\varphi ))$$

where *δ_h_* denotes the radial air-gap length under healthy operating conditions, and *e* is the eccentricity distance. The first-order approximation of the air-gap permeance can be expressed as follows:

(7) $$\Lambda (\varphi ,t)=\frac{1}{\delta (\varphi_{r},t)}=\frac{1}{\delta_{h}}\left[1+\varepsilon \cos(\frac{w_{s}}{p}t-\varphi )\right]$$

where *ε* represents the eccentricity degree, *ε* = *e*/*δ_h_*, and *w_s_* denotes the electrical angular velocity of the motor. According to Ampere’s law, the stator air-gap flux density *B_a_* of the motor can be expressed as follows:

(8) $$B_{a}(\varphi ,t)=\frac{\mu_{0}j_{0}}{\delta_{h}p}\cos(w_{s}t-\varphi )+\frac{\mu_{0}j_{0}\varepsilon }{2\delta_{h}p}\cos[(1\pm \frac{1}{p})w_{s}t-(p\pm 1)\varphi ]$$

Considering the effects of the motor winding structure and load conditions, the stator air-gap flux density *B_a_* can be expressed as follows:

(9) $$B_{a}(\varphi ,t)=\frac{\mu_{0}j_{0}}{\delta_{h}p}\cos(w_{s}t-\varphi )+\frac{\mu_{0}j_{0}\varepsilon }{2\delta_{h}p}\cos[(1\pm \frac{2n-1}{p})w_{s}t-(p\pm 1)\varphi ]$$

Equation (9) describes the variation in the stator air-gap flux density *B_a_* after the occurrence of a dynamic eccentricity fault in the motor, where *n* denotes a positive integer. The variation in *B_a_* introduces these harmonic components into the back electromotive force of the motor, which in turn gives rise to fault harmonics of specific frequencies in the stator current. The frequencies of these fault harmonics can be expressed as follows:

(10) $$f_{ec}=f_{e}(1\pm \frac{2n-1}{p})$$

where *f_e_* denotes the fundamental frequency of the motor.

Based on the above derivation, it can be seen that although both local demagnetization faults and eccentricity faults can cause distortion in the air-gap flux density distribution and further generate additional harmonics in the stator current, the mechanisms of harmonic generation and the corresponding frequency distribution patterns are different. To distinguish between these two types of faults, the characteristic harmonic frequencies corresponding to demagnetization faults and eccentricity faults are summarized in Table 1, thereby providing a theoretical basis for subsequent fault identification.

**Figure 1:**
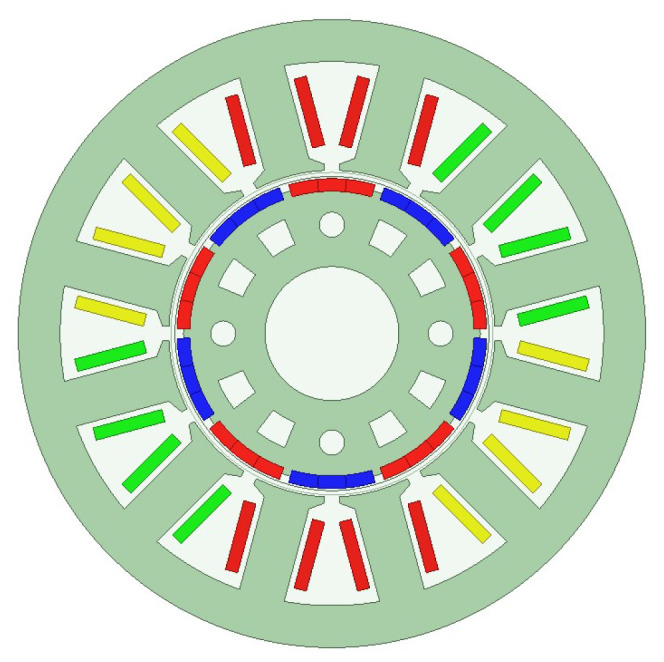
Structure of the PMSM.

To verify the foregoing analysis of the harmonic characteristics of local demagnetization faults and eccentricity faults, a 12-slot, 10-pole surface-mounted PMSM is taken as the object of study in this paper. The motor structure is shown in Figure 1.

**Figure 2:**
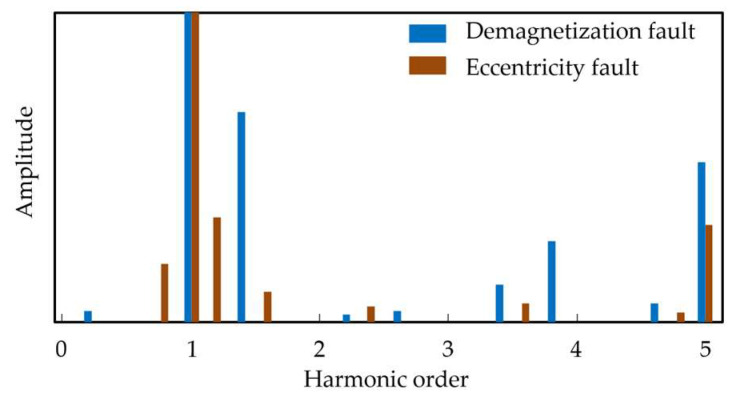
FFT frequency-domain spectra of different faults.

FFT is employed to perform frequency-domain analysis of the stator current signals under local demagnetization fault and eccentricity fault conditions. As shown in Figure 2, the harmonic frequencies associated with demagnetization faults are mainly distributed at the 1.4th, 3.4th, and 3.8th orders, whereas those associated with eccentricity faults are mainly distributed at the 0.8th, 1.2nd, and 1.6th orders. These results are consistent with the characteristic frequency sets of the two fault types listed in Table 1, and they also exhibit clear distinction in harmonic order distribution.

By comparing the current spectra under different fault conditions, the differences in frequency-domain characteristics between local demagnetization faults and eccentricity faults are verified. On this basis, when harmonic components corresponding to the characteristic frequencies of demagnetization faults appear in the current spectrum, the motor can be identified as operating under a local demagnetization fault condition.

### 2.3. Electromagnetic-Thermal Coupling Modeling

Frequency-domain analysis of stator current harmonics based on FFT can be used to identify demagnetization faults. However, if it is further applied to the quantitative detection of demagnetization severity, the harmonic amplitudes are susceptible to operating-condition variations, load fluctuations, and measurement noise, which imposes certain limitations. Therefore, in this paper, current harmonic features are used only for demagnetization fault identification.

On this basis, from the perspective of energy loss, once demagnetization occurs, the stator current amplitude increases in order to maintain the required electromagnetic torque output, thereby resulting in higher copper loss and an increase in winding temperature. Compared with current harmonic features, temperature signals, as integrated thermal-response quantities that reflect the motor operating condition, can provide a more stable indication of variations in demagnetization severity.

To characterize the variation law of electromagnetic-thermal coupling after demagnetization occurs in the motor, a demagnetization coefficient *k* ∈ [0, 1] is defined. The remanent flux density of the permanent magnet in the faulty pole and the permanent magnet flux linkage can then be expressed, respectively, as follows:

(11) $$Br^{\prime }=kBr$$

(12) $$\psi_{m}^{\prime }=k\psi_{m}$$

For a surface-mounted permanent magnet synchronous motor, under the vector control condition of *i_d_* = 0, the electromagnetic torque can be expressed as follows:

(13) $$T_{e}=\frac{3}{2}p\psi_{m}i_{q}$$

where *p* denotes the number of pole pairs, and *i_q_* represents the q-axis current.

When the load torque remains constant (*T_e_* = *T_L_*), the q-axis current required to maintain the same torque output after demagnetization can be expressed as follows:

(14) $${i_{q}}^{\prime }=\frac{T_{L}}{\frac{3}{2}p{\psi_{m}}^{\prime }}=\frac{1}{k}i_{q}$$

In the dq amplitude-invariant reference frame, the stator winding copper loss of the healthy motor can be expressed as follows:

(15) $$P_{cu}=\frac{3}{2}R_{s}{i_{q}}^{2}$$

When demagnetization occurs in the motor, the stator winding copper loss can be expressed as follows:

(16) $${P_{cu}}^{\prime }=\frac{3}{2}R_{s}({i_{q}}^{\prime })2=\frac{1}{k^{2}}P_{cu}$$

**Figure 3:**
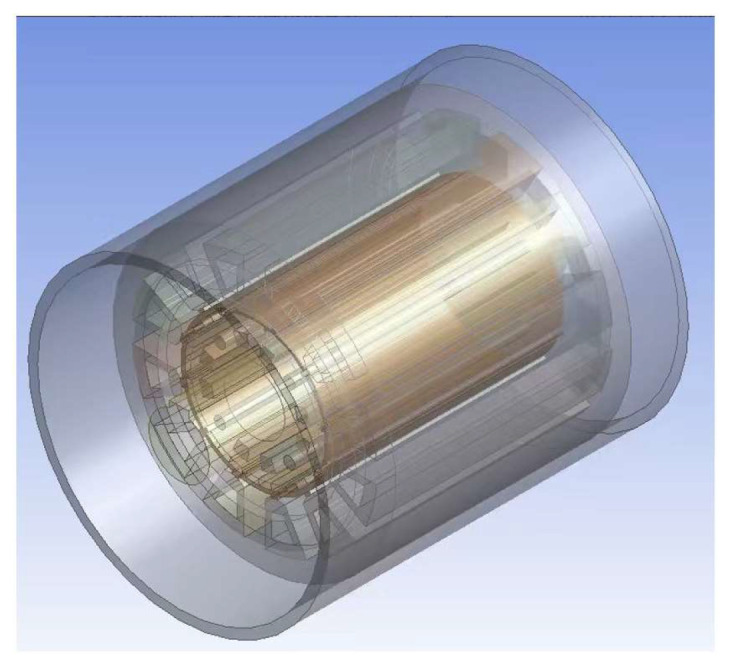
Three-dimensional structure of the permanent magnet synchronous motor.

According to (16), once demagnetization occurs in the motor, the winding copper loss increases, thereby leading to greater heat generation. Different levels of demagnetization affect the rate of internal heat accumulation in the motor, and thus produce distinguishable variation patterns in the winding temperature response. On this basis, an electromagnetic–thermal coupling simulation model is established on the ANSYS Workbench 2024 R1 platform to obtain winding temperature information under different demagnetization severities. Specifically, a motor model is established in Maxwell to calculate the loss distribution during operation. The loss results are then imported into the Transient Thermal module through Workbench and applied to the thermal analysis model as heat sources, while the corresponding thermal boundary conditions are imposed simultaneously. On this basis, the time-varying evolution of the motor temperature field is solved, the winding temperature at each time step is extracted, and a temperature time series is constructed in chronological order. The object of study is a 12-slot, 10-pole surface-mounted PMSM, whose three-dimensional structure is shown in Figure 3.

To further analyze the thermal response characteristics of the PMSM, it is necessary to comprehensively consider the motor losses and apply them to the thermal field model as heat sources, so as to characterize the heat generation power during motor operation. The winding copper loss can be calculated according to the relationship between current and resistance, and is expressed as follows:

(17) $$P=3I^{2}R$$

where *I* denotes the rms value of the stator current, and *R* represents the winding resistance.

When harmonic components and the rotating magnetization effect are taken into account, the rotating magnetization in the stator core can be equivalently modeled as an elliptical rotating magnetic field. This rotating magnetization process can be approximately described by two mutually orthogonal alternating magnetic field components, thereby enabling the actual magnetization state to be simulated. By separately calculating and superposing the iron-loss components caused by the fundamental magnetic field and each harmonic component, the total iron loss in the stator core can be obtained, and its expression is given as follows:

(18) $$\begin{array}{cc} P_{Fe} & =P_{h}+P_{c}+P_{e}\\ & =K_{h}f\sum_{k=0}^{\infty }k(B_{kmax}^{\alpha }+B_{kmin}^{\alpha })\\ & +k_{c}f^{2}\sum_{k=0}^{\infty }k^{2}(B_{kmax}^{2}+B_{kmin}^{2})\\ & +\frac{k_{e}}{{\left(2\pi \right)}^{3/2}}\frac{1}{T}{\int_{0}^{T}\left(\left|\frac{dB_{r}(t)}{dt}\right|\right)}^{1.5}+{\left|\frac{dB_{\theta }(t)}{dt}\right|}^{1.5})dt \end{array}$$

where *k_h_*, *k_c_*, and *k_e_* denote the hysteresis loss coefficient, eddy-current loss coefficient, and excess loss coefficient, respectively; *α* is the exponential parameter of hysteresis loss; *f* represents the alternating frequency of the magnetic field; and *k* denotes the harmonic order. *B_k_*_max_ and *B_k_*_min_ correspond to the major-axis flux density and minor-axis flux density of the *k*th harmonic, respectively. *B_r_*(*t*) and *B_θ_*(*t*) represent the radial and tangential flux densities inside the iron core, respectively.

Eddy-current loss is generated inside the permanent magnets, and this loss is converted into heat, thereby increasing the temperature of the permanent magnets. The eddy-current loss of the permanent magnets can be expressed as follows:

(19) $$P_{eddy}=\int_{V_{pm}}\frac{{\left|J_{e}\right|}^{2}}{\sigma }dV$$

where *V_pm_* denotes the volume of the permanent magnet, *σ* represents the electrical conductivity of the permanent magnet, and *J_e_* is the current density within the permanent magnet.

**Figure 4:**
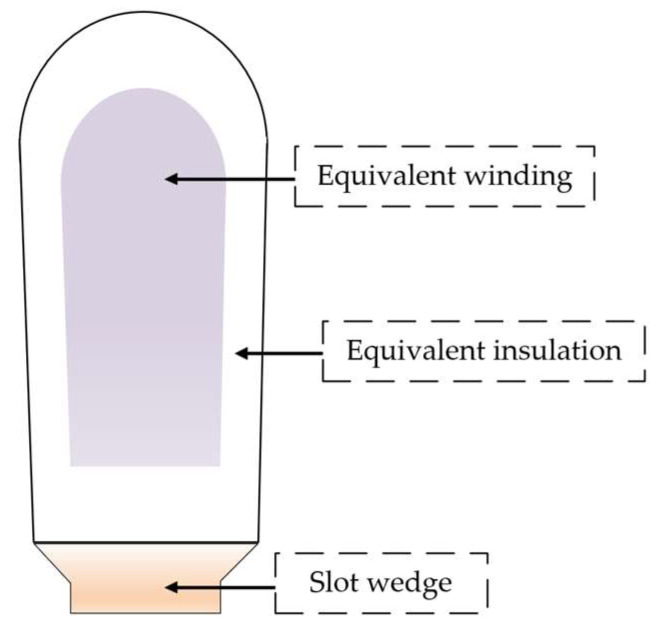
Equivalent model of the stator slot.

The motor stator slots are composed of copper conductors and multiple layers of insulating materials wrapped around them. To reduce model complexity and improve computational efficiency, the internal structure of the stator slots is treated equivalently in this paper. The copper conductors inside each slot are regarded as an integral body, and the insulating materials are assumed to be uniformly distributed around them, while the influence of local temperature gradients inside the stator slots is neglected. Figure 4 illustrates the equivalent model of the stator slot.

Based on the thermal parameters of the various insulating materials within the slot, the thermal conductivity of the equivalent insulation can be obtained, and its expression is given as follows:

(20) $$\lambda_{eq}=\frac{\sum_{i=1}^{n}\delta_{i}}{\sum_{i=1}^{n}\frac{\delta_{i}}{\lambda_{i}}}$$

where *λ_eq_* denotes the thermal conductivity of the equivalent insulation, *δ_i_* is the thickness of the equivalent insulation material, and *λ_i_* represents the average thermal conductivity of the insulating materials.

The thermal conductivity of the motor core can be analyzed separately along the radial, circumferential, and axial directions. Since the core is formed by stacking multiple silicon steel laminations along the axial direction, with insulating coatings between adjacent laminations, its heat conduction exhibits pronounced anisotropy. During axial heat transfer, the heat flow passes successively through the silicon steel sheets and insulation layers, which can be equivalently modeled as a multi-layer series heat transfer structure. In contrast, during radial and circumferential heat transfer, the materials of each layer are distributed in parallel along the heat transfer path, which can be equivalently modeled as a multi-layer parallel heat transfer structure. Based on this, the expressions for the equivalent thermal conductivity in each direction can be obtained as follows:

(21) $$\left\{\begin{array}{l} \lambda_{x}=\lambda_{y}=\frac{\delta_{Fe}\lambda_{1}+\delta_{0}\lambda_{0}}{\delta_{Fe}+\delta_{0}}=K_{Fe}\lambda_{1}+(1-K_{Fe})\lambda_{0}\\ \lambda_{z}=\frac{\delta_{Fe}+\delta_{0}}{\frac{\delta_{Fe}}{\lambda_{1}}+\frac{\delta_{0}}{\lambda_{0}}}=\frac{1}{\frac{K_{Fe}}{\lambda_{1}}+\frac{\left(1-K_{Fe}\right)}{\lambda_{0}}} \end{array}\right.$$

where *λ_x_*, *λ_y_*, and *λ_z_* denote the equivalent thermal conductivities of the core in the radial, circumferential, and axial directions, respectively; *δ_Fe_* and *δ*_0_ represent the net lengths of the silicon steel sheet and the insulating medium, respectively; *λ*_1_ and *λ*_0_ denote the thermal conductivities of the silicon steel sheet and the insulating medium, respectively; and *K_Fe_* is the stacking factor of the core.

In the thermal analysis model, corresponding convective heat transfer boundary conditions are also specified for the outer surface of the housing, the winding end regions, the stator end regions, and the rotor end regions, so as to characterize the heat exchange process between the motor and the surrounding environment during operation. Under the combined effects of the above heat-source distribution, heat-conduction paths, and heat-dissipation boundaries, the temperature field can be solved, thereby yielding the winding temperature at different operating instants.

### 2.4. Analysis of Temperature Response Characteristics

After the temperature field is solved, the influence mechanism of local demagnetization on the variation characteristics of the motor thermal response can be investigated by analyzing the evolution of winding temperature with operating time. In the simulation, the initial ambient temperature is set to 16 °C, and the motor operates continuously under rated operating conditions. The temperature field distribution at 100 min of operation is selected as the analysis instant, and the temperature data of the motor winding are extracted to provide a basis for the subsequent analysis of temperature responses under healthy and demagnetized conditions.

**Figure 5:**
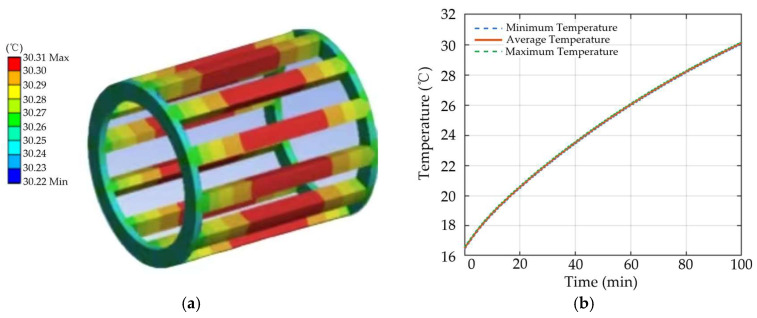
Healthy motor: (**a**) three-dimensional winding temperature distribution; (**b**) winding temperature curve.

As shown in Figure 5, the heat generation in the healthy motor is mainly concentrated in the stator winding. The winding temperature gradually increases with operating time and then tends to become stable, indicating that the system progressively approaches thermal equilibrium. This result provides a reference for the subsequent analysis of temperature responses under demagnetization fault conditions.

**Figure 6:**
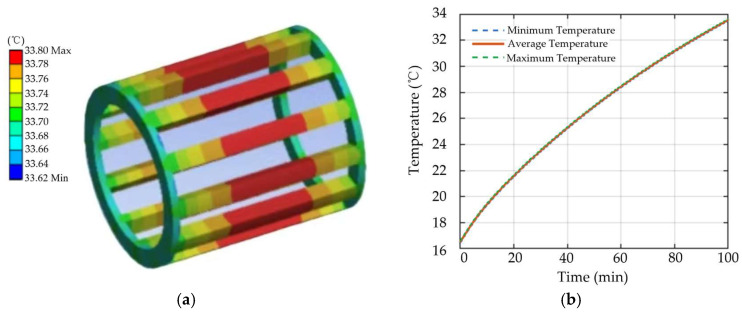
Local demagnetization motor: (**a**) three-dimensional winding temperature distribution; (**b**) winding temperature curve.

As shown in Figure 6, when a local demagnetization fault occurs in the motor, the excitation capability of the faulty pole decreases, leading to distortion in the air-gap flux density distribution and a reduction in torque production capability. To maintain the required load torque output, the control system must increase the stator current to compensate for the torque reduction, which in turn causes an increase in winding copper loss. Compared with the healthy condition, the slope of the winding temperature rise curve becomes steeper under demagnetization fault conditions, indicating a faster rate of temperature increase over time and reflecting the intensifying effect of demagnetization on the thermal response process.

To further investigate the influence of different demagnetization severities on temperature response, four demagnetization levels of a single magnetic pole, namely 25%, 50%, 75%, and 100%, are imposed in the finite element model, and the corresponding winding temperature variation curves are extracted under the same operating condition.

**Figure 7:**
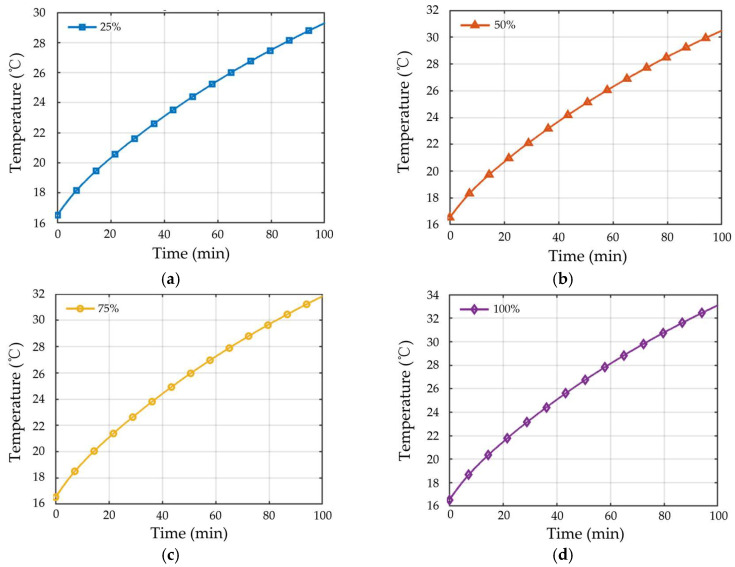
Winding temperature rise under different demagnetization levels: (**a**) 25% demagnetization, (**b**) 50% demagnetization, (**c**) 75% demagnetization, and (**d**) 100% demagnetization.

As shown in Figure 7, the evolution of winding temperature exhibits distinct behaviors under different demagnetization severities. When the demagnetization severity reaches 100%, the magnetic properties of the permanent magnet are significantly degraded, leading to a marked increase in motor energy loss, as reflected by the fastest rise in winding temperature over time. As the demagnetization severity decreases to 75% and 50%, the magnetic properties of the permanent magnet are not completely lost, but both the amplitude and spatial distribution of the air-gap magnetic field have deteriorated to different extents, resulting in lower temperature response levels than those observed under complete demagnetization. In contrast, when the demagnetization severity is 25%, the degradation in magnetic properties is relatively limited, and its influence on the motor operating condition is comparatively small. Accordingly, the variation trend of winding temperature remains at a relatively low level.

**Figure 8:**
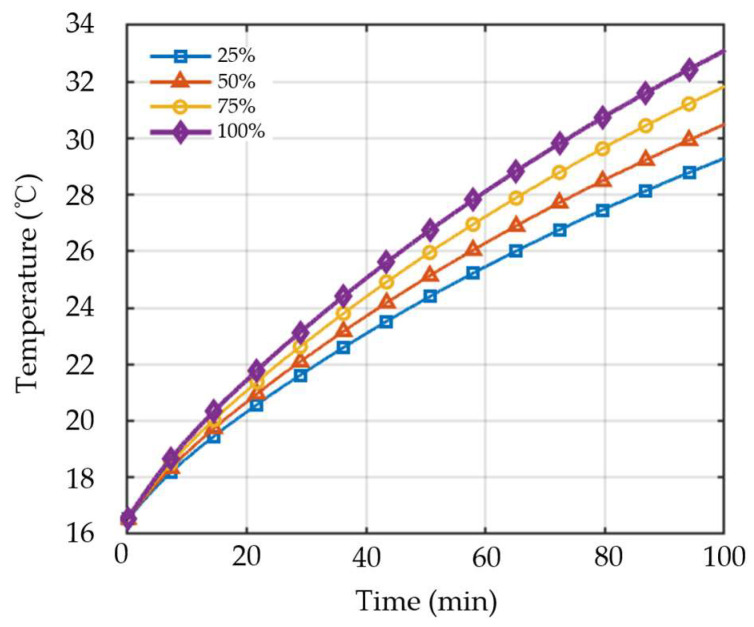
Comparison of winding temperature rise under different demagnetization levels.

Figure 8 compares the winding temperature rise under different demagnetization severities. The results indicate that the winding temperature time series contains effective information capable of characterizing differences in demagnetization severity, thereby providing a physical basis for the subsequent temperature-signal-based demagnetization severity detection method.

## 3. Demagnetization Severity Detection Method Based on Temperature Signal Under Multiple Operating Conditions

**Figure 9:**
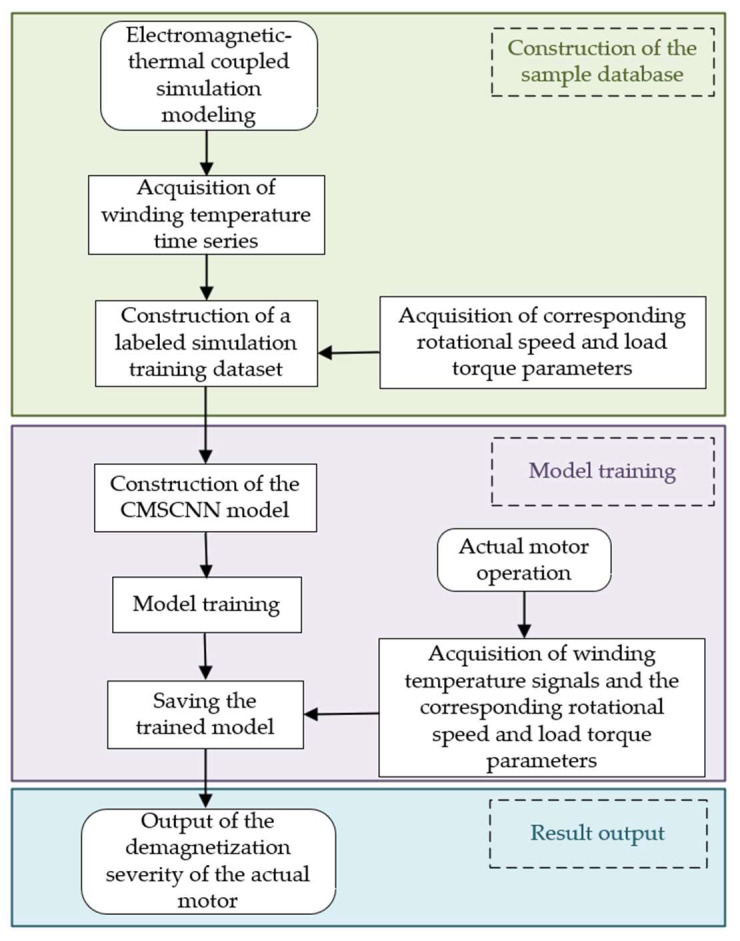
Overall flowchart.

To achieve accurate detection of motor demagnetization severity under multiple operating conditions, this paper proposes a demagnetization severity detection method based on temperature signal and Convolutional Neural Network. Considering that the temperature response is influenced not only by demagnetization severity but also closely related to operating conditions, the temperature time series, speed, and load torque are jointly constructed into a three-dimensional state space to characterize the motor operating condition. Subsequently, CMSCNN is employed to extract features from the temperature sequence, thereby establishing the mapping relationship between the three-dimensional state space and demagnetization severity. The overall framework is shown in Figure 9, and the specific steps are as follows:

(1) Based on the electromagnetic–thermal coupling model, simulations are conducted under different demagnetization severities and different operating conditions characterized by speed and load torque, so as to obtain winding temperature time-series data that evolve over time.
(2) Speed and load torque are introduced into the model as operating-state variables, and a three-dimensional state space composed of the temperature time series, speed, and load torque is established. On this basis, a data sample set is constructed.
(3) CMSCNN is introduced to extract features from the temperature time series. Speed and load torque are incorporated as conditional variables to modulate the temperature signal, thereby establishing the mapping relationship between temperature response and demagnetization severity under multiple operating conditions.
(4) The model is trained, and the trained model is then saved.
(5) Temperature signals and the corresponding operating condition information are collected in real time from the practical motor during operation and used as inputs to the model, thereby enabling demagnetization severity detection.

### 3.1. Mapping Relationship Between the Three-Dimensional State Space and Demagnetization Severity

Motor temperature is influenced not only by demagnetization severity, but also closely related to operating conditions and operating time. Variations in temperature under different operating conditions may therefore interfere with demagnetization severity detection.

**Figure 10:**
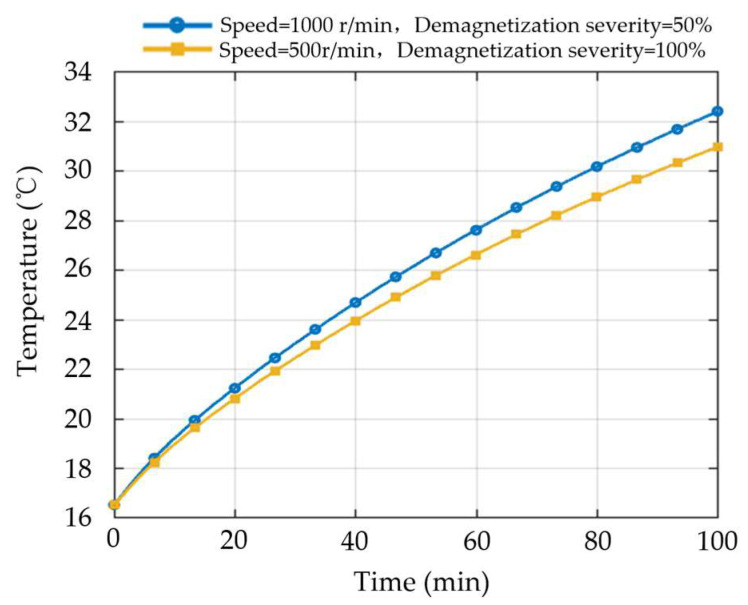
Comparison of winding temperature rise curves under different speeds for 50% and 100% demagnetization levels.

To investigate how variations in speed affect the relationship between temperature response and demagnetization severity, the winding temperature variation curves corresponding to demagnetization severities of 50% and 100% under different speeds are compared while keeping the load torque constant. The results are shown in Figure 10. It can be observed that the winding temperature under the condition of lower demagnetization severity but higher speed is higher than that under the condition of higher demagnetization severity but lower speed, indicating that the influence of speed on temperature exceeds that of demagnetization severity itself. This phenomenon shows that, under multi-speed operating conditions, the temperature response is not determined solely by demagnetization severity, but rather results from the coupled effect of operating conditions and demagnetization state. Therefore, when the range of speed variation is large, demagnetization severity detection based only on temperature amplitude or temperature rise level will make it difficult to distinguish temperature signals corresponding to different demagnetization severities, thereby reducing the accuracy of temperature signal-based demagnetization severity detection under multiple operating conditions.

**Figure 11:**
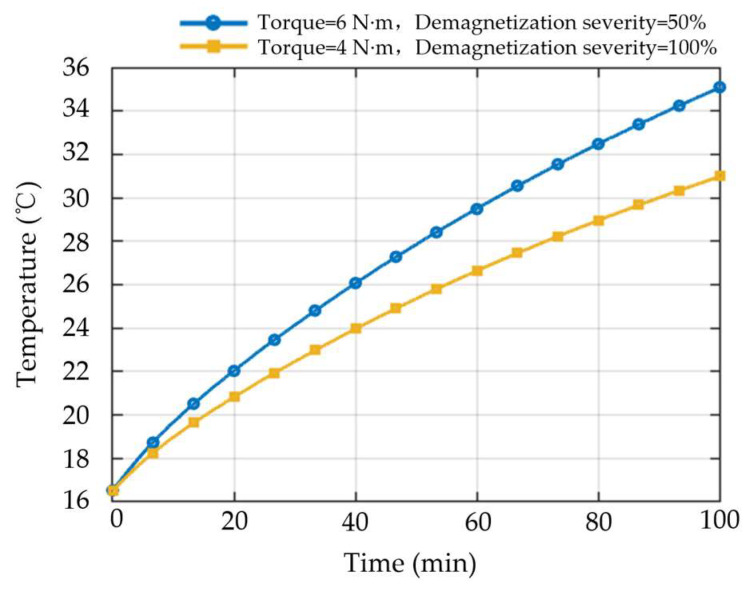
Comparison of winding temperature rise curves under different load torque conditions for 50% and 100% demagnetization levels.

To further analyze the effect of load torque variation on the temperature response, the winding temperature variation curves at demagnetization severities of 50% and 100% under different torque conditions are compared at the same speed. As shown in Figure 11, the temperature under the condition of higher load torque but lower demagnetization severity is higher than that under the condition of lower load torque but higher demagnetization severity. This phenomenon indicates that the influence of load torque variation on temperature exceeds that of demagnetization severity itself. These results further demonstrate that, under complex operating conditions involving multiple speeds and multiple torques, it is difficult to stably characterize the variation in demagnetization severity by relying solely on a single temperature signal.

In addition to operating conditions, operating duration is also an important factor affecting temperature variation. As the operating time increases, heat continuously accumulates inside the motor, leading to a sustained rise in winding temperature. Even when the speed and load torque remain constant, the corresponding temperatures still differ under different operating durations. Therefore, even at the same demagnetization severity, the temperature response may vary with operating duration.

**Figure 12:**
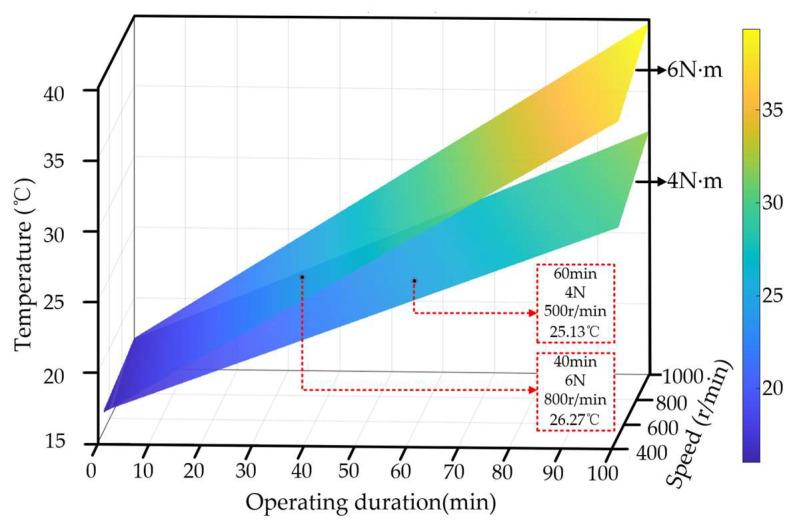
Three-dimensional plot of the effects of speed, load torque, and operating duration on winding temperature.

As shown in Figure 12, to investigate the combined effects of speed, load torque, and operating duration on the temperature response, a three-dimensional analysis is conducted on the winding temperature variation under the same demagnetization severity. The results show that the winding temperature is jointly influenced by speed, torque, and operating duration. Different operating conditions may, to some extent, amplify or mask the effect of demagnetization faults on the temperature rise process, making the temperature signals under different demagnetization severities difficult to distinguish. Therefore, it is necessary to jointly model the temperature signal and operating condition variables to improve the accuracy of demagnetization severity detection.

The three-dimensional state space constructed in this paper is based on the above joint state representation. Specifically, during sample construction, the temperature time series, rotational speed, and load torque corresponding to the same sample are organized into one input group to jointly describe the motor operating state. In this way, the coupling relationship among demagnetization severity, operating conditions, and temperature response can be incorporated into a unified representation framework, thereby alleviating the insufficient representation capability of temperature information alone under multiple operating conditions.

### 3.2. Conditionally Modulated Multi-Scale Convolutional Neural Network

**Figure 13:**
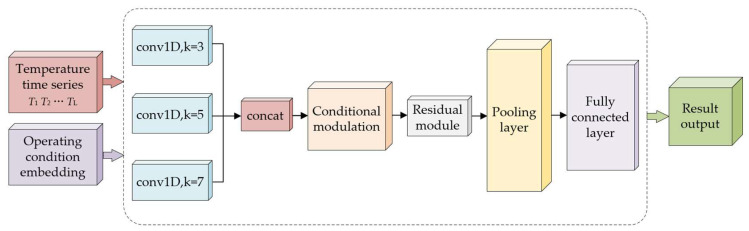
Schematic diagram of the CMSCNN.

In this section, based on the input of the temperature time series, speed and load torque are introduced as operating condition variables, and the operating condition information is incorporated into the feature extraction process through a conditional modulation mechanism. Meanwhile, multi-scale convolution and a residual structure are combined to enhance the model’s ability to extract multi-time-scale information from the temperature sequence, thereby constructing CMSCNN for demagnetization severity detection under multiple operating conditions. Figure 13 shows the schematic diagram of the proposed model.

**Figure 14:**
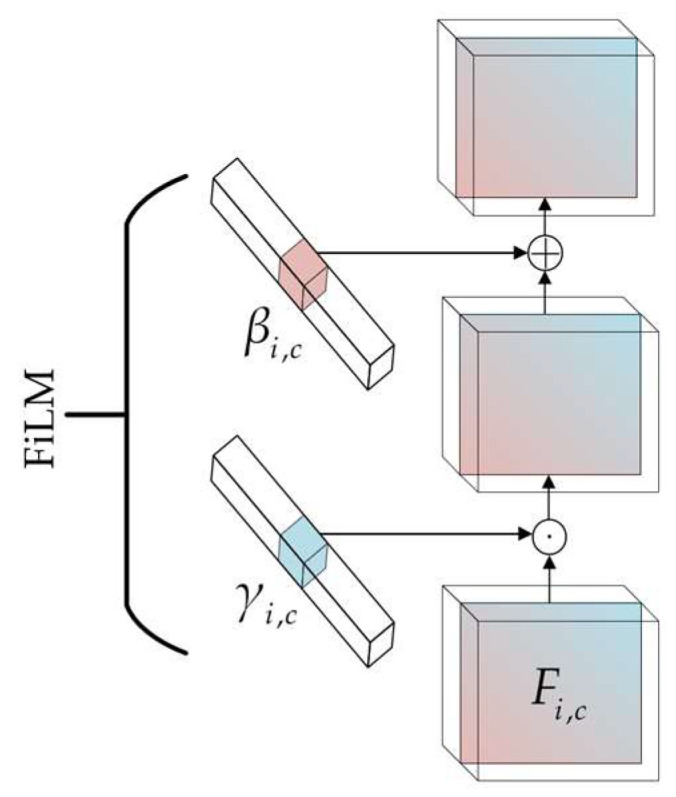
Schematic diagram of the FiLM mechanism.

To achieve adaptive regulation of the temperature feature extraction process by operating-condition information, Feature-wise Linear Modulation (FiLM) is introduced to modulate the intermediate features. Figure 14 shows a schematic diagram of the FiLM mechanism. Let the input feature be *F_i,c_*, where *i* denotes the feature position and *c* denotes the channel index. The modulation process can be expressed as follows:

(22) $${\hat{F}}_{i,c}=\gamma_{i,c}F_{i,c}+\beta_{i,c}$$

where *γ_i,c_* and *β_i,c_* denote the modulation coefficients generated from the conditional information, respectively.

This mechanism maps speed and load torque into modulation parameters, enabling the temperature features to be adaptively adjusted according to the operating conditions. By regulating the channel response intensity, it suppresses feature shifts caused by operating condition variations and highlights demagnetization features that remain consistent under different operating conditions.

**Figure 15:**
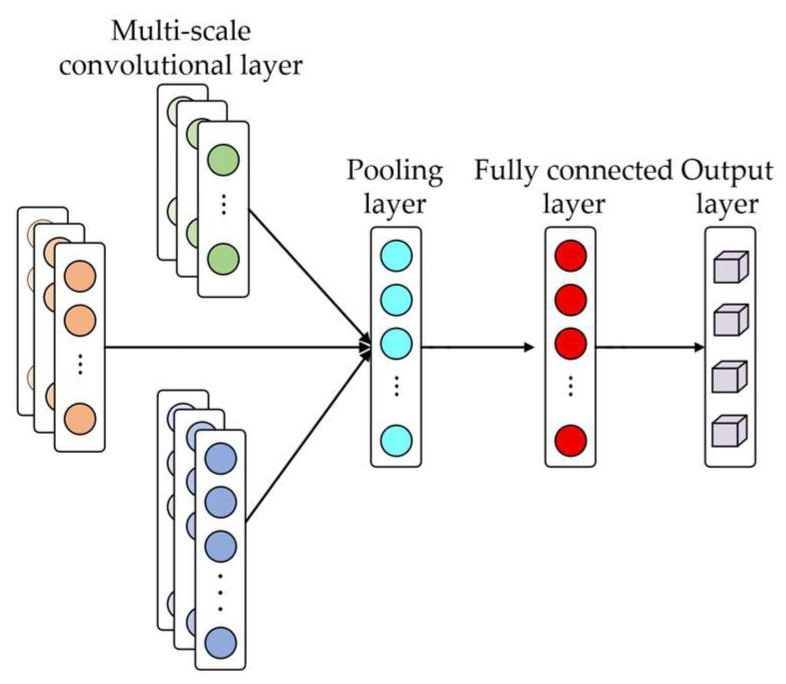
Schematic diagram of multi-scale convolution.

Considering that the temperature time series exhibits multi-time-scale characteristics, a multi-scale convolution structure is adopted in this paper for feature extraction. As shown in Figure 15, this structure processes the input sequence through multiple parallel convolution branches with convolution kernels of different sizes, so as to extract feature information from different receptive field ranges. Smaller convolution kernels focus on local fluctuations and fine-grained variations, whereas larger convolution kernels emphasize the overall variation trend. By fusing the responses of multi-scale convolutions, the model’s capability to extract complex temporal features can be enhanced, thereby improving the performance of demagnetization severity detection.

**Figure 16:**
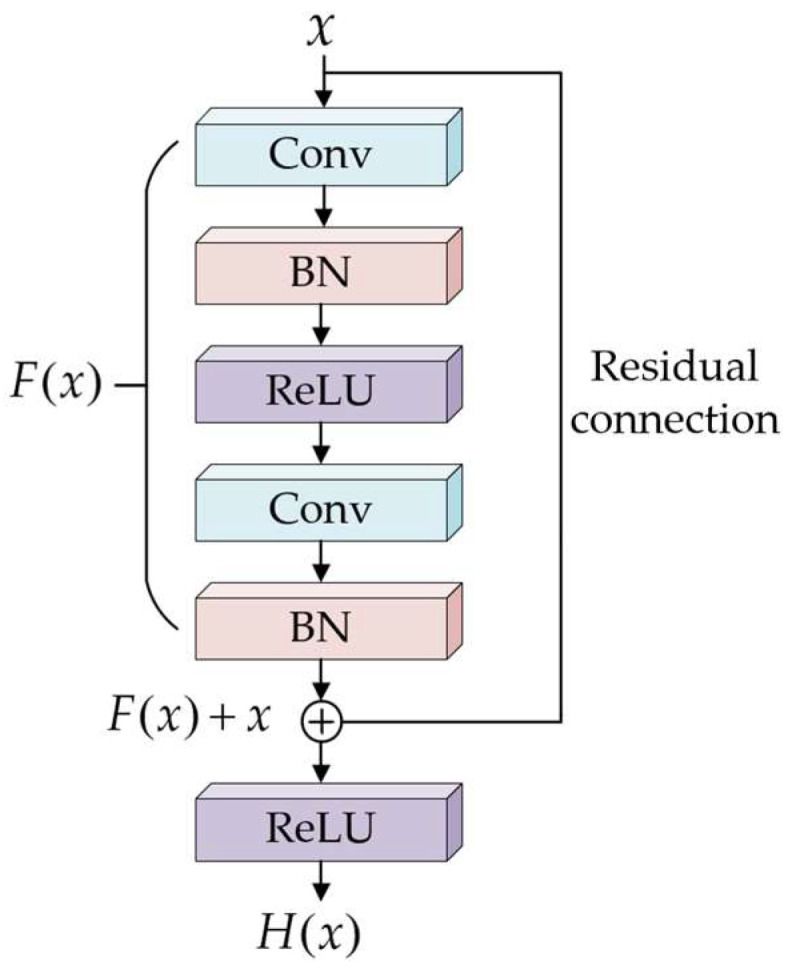
Schematic diagram of the residual structure.

To alleviate problems such as gradient vanishing and network degradation that may arise during the training of deep networks, a residual structure is further introduced in this paper, as shown in Figure 16. The residual block consists of two convolutional layers, batch normalization (BN) layers, and activation layers. The computation process of the residual block can be expressed as follows:

(23) H(x)=F(x)+x

where *x* denotes the input to the residual block, and *H*(*x*) represents the desired output function.

In terms of network parameter settings, the model takes the temperature time series as the primary input, while speed and load torque are used as conditional inputs. The network uses convolutional layers to extract temporal features, with conditional information incorporated for feature modulation. The detailed network parameters are listed in Table 2.

During model training, the sample set is divided into the training set, validation set, and test set according to a specified ratio. The Adam optimizer is employed for training, and parameter updates are performed in conjunction with a learning rate decay strategy. The cross-entropy loss function is adopted as the objective function, and the optimal parameters are selected based on the performance on the validation set. The specific training parameters are listed in Table 3.

### 3.3. Local Demagnetization Severity Detection

After model training is completed, the test set is used to evaluate the demagnetization severity detection performance of the proposed CMSCNN. The prediction accuracies for samples with different demagnetization severities are listed in Table 4. As shown in Table 4, the detection accuracies for all four groups of demagnetization severity samples remain above 97%, with an average detection accuracy of 98.06%, indicating that the proposed CMSCNN can accurately identify different demagnetization severities.

To validate the effectiveness of the proposed method, baseline CNN, support vector machine (SVM), and long short-term memory (LSTM) were selected as comparative methods. All models used the same input data, with each sample jointly constructed from the temperature time series, rotational speed, and load torque, as follows:

(1) CMSCNN: By introducing multi-scale convolution, conditional modulation, and residual modules, feature extraction capability and classification discrimination ability are enhanced.
(2) Baseline CNN: A basic Convolutional Neural Network architecture is adopted to perform feature learning and classification tasks.
(3) Long short-term memory (LSTM): This is a typical deep learning method for time-series analysis, which performs classification by utilizing sequence modeling capability.
(4) Support vector machine (SVM): This is a traditional machine learning classification method, which performs category discrimination by constructing classification boundaries.

According to the detection results in Table 5, the proposed CMSCNN achieved the highest detection accuracy, while the baseline CNN achieved the second-highest accuracy among all comparative methods. Therefore, to further analyze the classification performance of different models, the proposed CMSCNN and the baseline CNN were selected for comparison.

**Figure 17:**
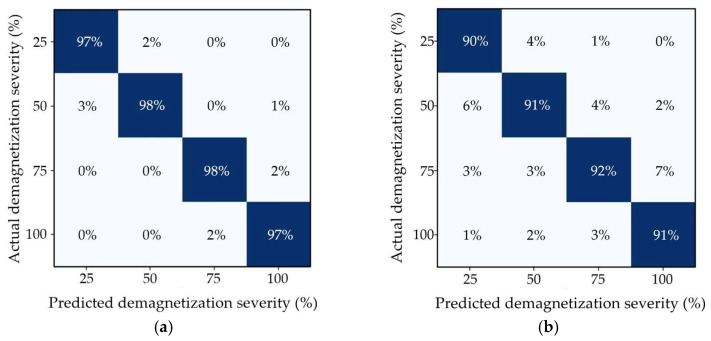
Confusion matrices: (**a**) CMSCNN model; (**b**) baseline CNN model.

As shown by the confusion matrices in Figure 17, the proposed CMSCNN model outperforms the baseline CNN model in terms of overall classification accuracy. In the confusion matrix of the baseline CNN model, a certain degree of misclassification can be observed among different demagnetization severity categories, especially between adjacent demagnetization levels. By contrast, the recognition results of the proposed CMSCNN model are more concentrated for each demagnetization category, and the diagonal elements are more prominent, indicating that its classification performance is more accurate and stable.

**Figure 18:**
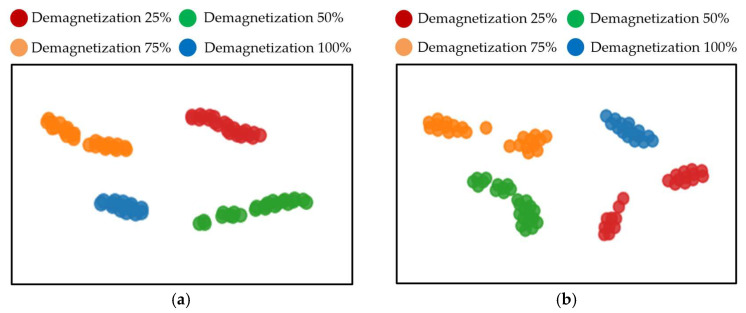
t-SNE visualization results: (**a**) CMSCNN model; (**b**) baseline CNN model.

Further analysis based on the t-SNE visualization results, as shown in Figure 18, indicates that the baseline CNN model exhibits relatively scattered intra-class distributions and small inter-class distances. In contrast, the features extracted by the proposed CMSCNN model are more compactly distributed and show higher inter-class separability.

The results indicate that the proposed CMSCNN has advantages over the baseline CNN in both feature extraction and classification discrimination. The multi-scale convolution can extract demagnetization-related features from the temperature sequence at different temporal scales. By incorporating operating-condition information such as rotational speed and load torque, the conditional modulation mechanism enables the network to learn demagnetization features that remain consistent under different operating conditions. Meanwhile, the residual structure helps alleviate the gradient degradation problem during the training of deep networks, thereby improving the model’s capability for demagnetization severity detection.

To evaluate the detection robustness of the proposed method under noise interference, Gaussian white noise with signal-to-noise ratios of 20 dB, 10 dB, and 5 dB is added to the test set, respectively, and the trained model is then used to perform demagnetization severity detection. The results are listed in Table 6. It can be observed that, although the detection accuracies of both methods decrease as the signal-to-noise ratio declines, the proposed CMSCNN model still achieves a detection accuracy of 92.07% even under strong noise interference at 5 dB. This result indicates that the proposed method can suppress the influence of noise on temperature feature extraction and thus exhibits good robustness.

## 4. Experimental Results and Analysis

**Figure 19:**
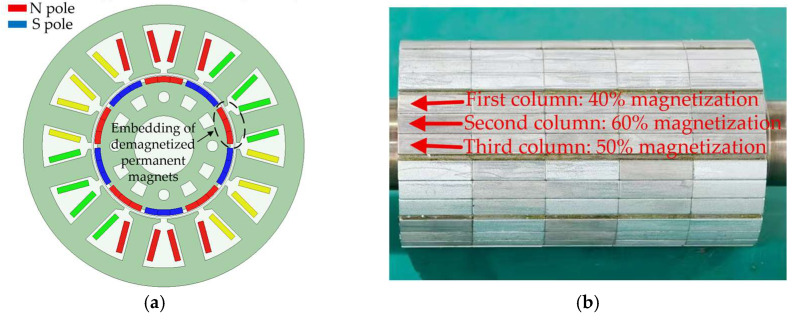
(**a**) Location of the demagnetized permanent magnet; (**b**) schematic diagram of partitioned magnetization of the magnetic pole.

**Figure 20:**
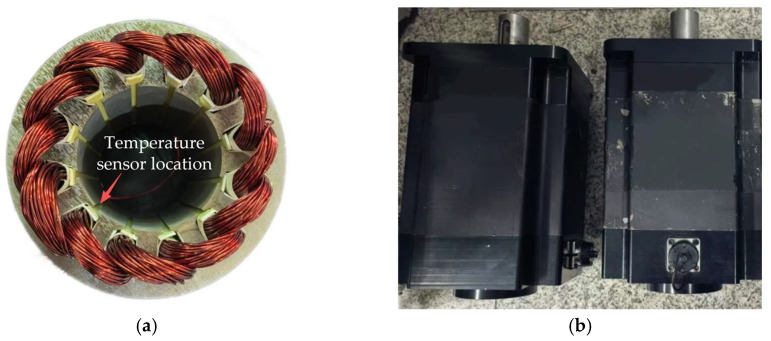
(**a**) Location of the temperature sensor; (**b**) experimental prototype.

To verify the effectiveness of the proposed method, two prototype motors were fabricated, in which M1 was a healthy motor and M2 was a motor with 50% local demagnetization. As shown in Figure 19, unsaturated magnetization was implemented using a magnetizing machine during the fabrication of the faulty prototype. To equivalently simulate 50% demagnetization in a single magnetic pole, the three columnar segments of the permanent magnet in that pole were set to different magnetization levels. This magnetization strategy was intended to avoid simplifying the fault as whole-pole demagnetization, thereby enabling the faulty prototype to reflect the spatial asymmetry effect caused by demagnetization. As shown in Figure 20, in terms of temperature measurement, a PT100 temperature sensor (Heraeus, Hanau, Germany) is used to measure the stator winding temperature, and the temperature signal is acquired and stored by a TP9000 multi-channel data logger (Shenzhen Toprie Electronics Co., Ltd., Shenzhen, China), thereby obtaining the temperature time series during the actual motor operation. The PT100 temperature sensor is a platinum resistance temperature sensor, with a resistance of 100 Ω at 0 °C, and its resistance changes with temperature in a relatively stable and regular manner. It has the advantages of high measurement accuracy, good stability, and strong anti-interference capability. Table 7 presents the parameters of 12 slots, 10 poles permanent magnet synchronous motors.

**Figure 21:**
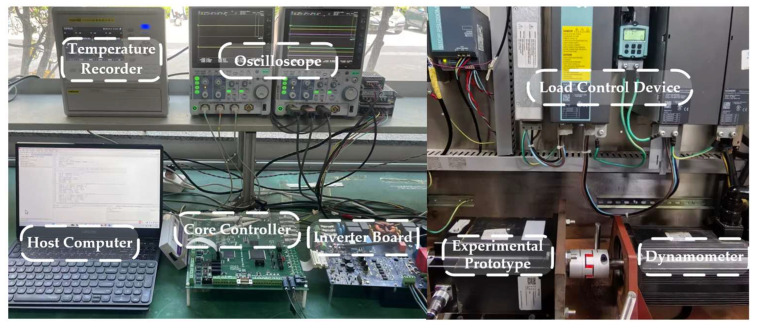
Experimental platform.

The experimental platform for demagnetization fault diagnosis of the permanent magnet synchronous motor mainly includes temperature recorder, oscilloscope, host computer, core controller, inverter board, experimental prototype, load control device S120 (Siemens AG, Munich, Germany), and dynamometer. The host computer is used for control program downloading, parameter setting, and experimental process monitoring. The core controller consists of DSP and FPGA, where DSP is responsible for the real-time execution of control algorithms such as current loop and speed loop, while FPGA is responsible for high-speed logic control and timing management. Both the sampling frequency and the switching frequency of the control system are 10 kHz. The inverter is used to drive the experimental prototype. The experimental prototype is connected to the dynamometer, and the load control device cooperates with the dynamometer to achieve precise adjustment of load torque under different operating conditions. The temperature signal is acquired and stored by the TP9000 multi-channel data recorder. Figure 21 shows the experimental platform.

**Figure 22:**
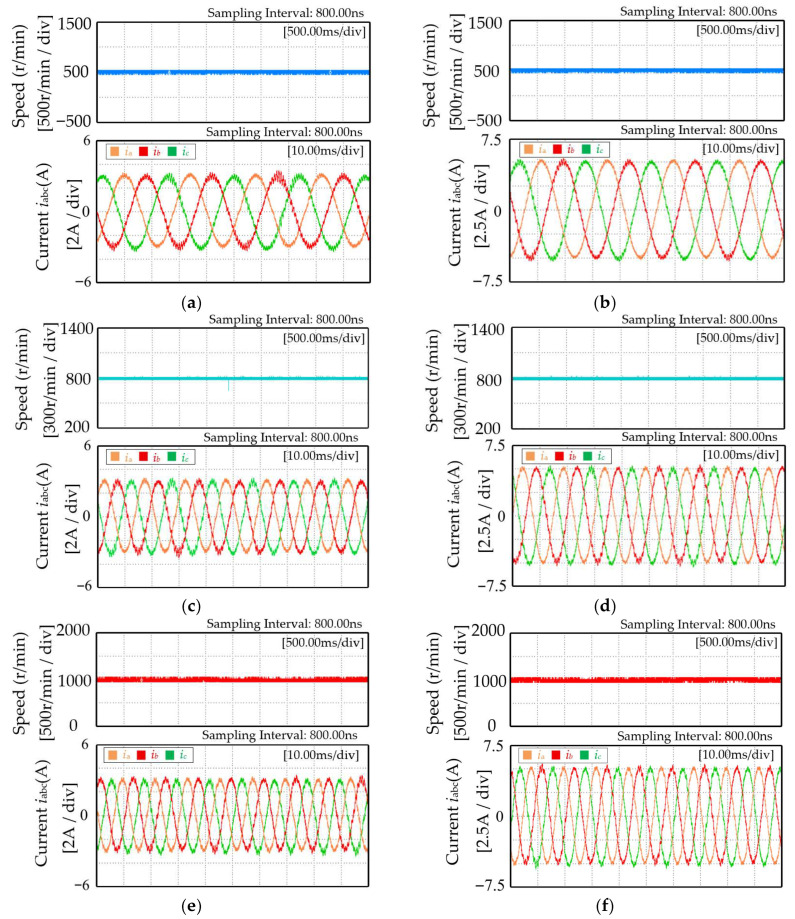
Experimental waveforms under different combinations of speeds and load torques: (**a**) 500 r/min, 4 N·m; (**b**) 500 r/min, 6 N·m; (**c**) 800 r/min, 4 N·m; (**d**) 800 r/min, 6 N·m; (**e**) 1000 r/min, 4 N·m; and (**f**) 1000 r/min, 6 N·m.

Temperature acquisition experiments are conducted on the demagnetized prototype under different combinations of speed and load torque. The specific operating conditions for data acquisition are listed in Table 8. Figure 22 shows the waveforms under different speeds and the electrical signals under different load torque conditions.

**Figure 23:**
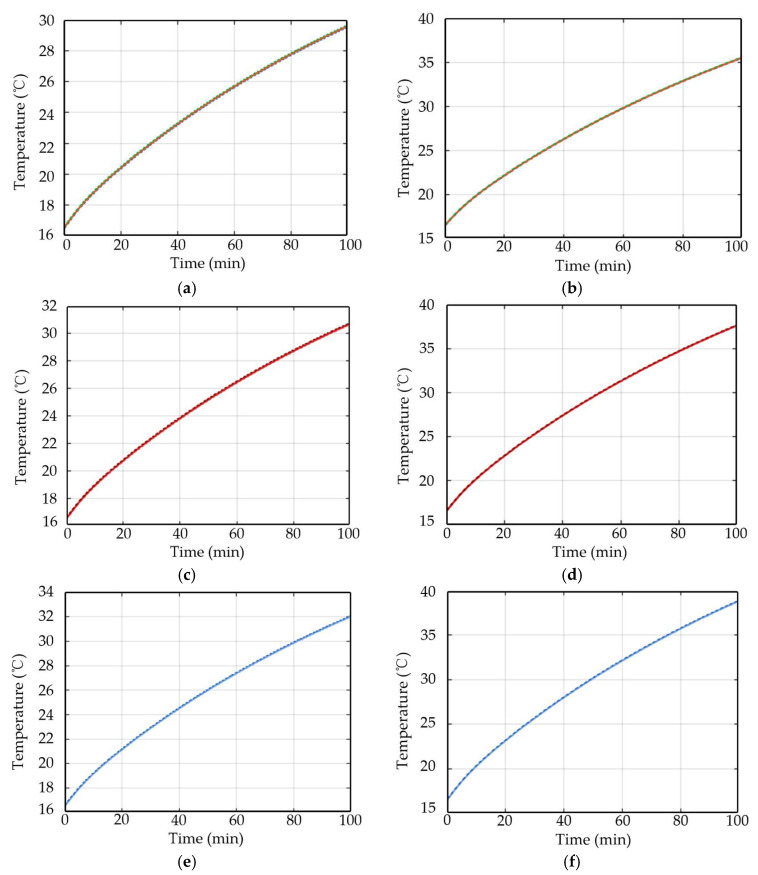
Winding temperature rise curves under different combinations of speeds and load torques: (**a**) 500 r/min, 4 N·m; (**b**) 500 r/min, 6 N·m; (**c**) 800 r/min, 4 N·m; (**d**) 800 r/min, 6 N·m; (**e**) 1000 r/min, 4 N·m; and (**f**) 1000 r/min, 6 N·m.

The winding-end temperature variation curves over time are collected under different speed and load torque conditions, and the experimental results are shown in Figure 23. It can be observed from the figure that the temperature evolution over time varies under different operating conditions. As the speed increases or the load torque rises, the winding temperature increases more rapidly and reaches higher values accordingly. These phenomena indicate that variations in operating conditions alter the distribution of electromagnetic losses and the rate of heat accumulation inside the motor, thereby affecting the temperature response characteristics of the windings. This experimental result is consistent with the foregoing theoretical analysis based on electromagnetic–thermal coupling, and it verifies the influence of operating conditions on the temperature variation process.

Based on CMSCNN proposed in this paper, demagnetization severity detection is performed on the measured data collected from the faulty prototype. Table 9 presents the detection results of demagnetization severity based on the measured temperature signals under six groups of operating conditions. The statistical metric is defined as the proportion of test samples that are classified by the model as having a demagnetization severity of 50%. As shown in Table 9, under different combinations of speed and load torque, the detection accuracy remains above 92%, with an average detection accuracy of 93.34%. These results indicate that the model is still able to maintain stable output when the operating conditions vary. This further demonstrates that, by jointly modeling operating-condition information and temperature signals, the disturbance caused by operating-condition variations to the temperature response is mitigated, allowing the model to learn demagnetization severity discrimination features that are invariant to operating conditions. The above results verify the effectiveness of the proposed method when applied to measured temperature signals.

## 5. Conclusions

This paper proposes a local demagnetization severity detection method for permanent magnet synchronous motors based on temperature signal and Convolutional Neural Network. First, the differences between local demagnetization and eccentricity fault in stator current harmonics are analyzed from an electromagnetic perspective, and fast Fourier transform (FFT) is used for frequency-domain analysis of the stator current to identify demagnetization faults. Second, an electromagnetic-thermal coupling model is established to obtain the winding temperature response under different demagnetization severities and operating conditions, while taking motor losses and heat dissipation boundary conditions into account. Further analysis based on electromagnetic torque and loss mechanisms shows that, after demagnetization occurs, the motor needs to increase the stator current in order to maintain the required output torque, which in turn leads to increased copper loss and accelerates the winding temperature rise process, thereby revealing the intrinsic relationship between temperature response and demagnetization severity. On this basis, the temperature time series, speed, and load torque are jointly constructed into a three-dimensional state space, and CMSCNN is employed to establish the mapping relationship between the three-dimensional state space and demagnetization severity. Experimental results show that the proposed method can maintain high demagnetization severity detection accuracy under multiple operating conditions, thereby verifying its effectiveness.

## Figures and Tables

**Table 1:** Comparison of different fault harmonic frequencies.

	Demagnetization Fault	Eccentricity Fault
Fault harmonics	$f_{e}(1\pm \frac{n}{p})$	$f_{e}(1\pm \frac{2n-1}{p})$

**Table 2:** Network parameter settings.

Parameter Name	Parameter Setting	Parameter Name	Parameter Setting
Input sequence length	3500	Number of channels	16
Conditional input dimension	2	FiLM hidden-layer dimension	32
Number of multi-scale branches	3	Residual kernel size	3
Convolution kernel size	3, 5, 7	Pooling type	Average pooling

**Table 3:** Model training parameter settings.

Parameter Name	Parameter Setting	Parameter Name	Parameter Setting
Dataset split	8:1:1	Initial learning rate	0.001
Batch size	16	Weight decay coefficient	1 × 10^−4^
Epoch	60	Learning rate decay strategy	StepLR
Optimizer	Adam	Step size	20
Loss function	Cross-Entropy loss	Decay factor	0.5

**Table 4:** Demagnetization severity detection accuracy on the test set.

	Group 1	Group 2	Group 3	Group 4
Accuracy	97.35%	98.74%	98.52%	97.61%

**Table 5:** Average demagnetization severity detection accuracy of different methods.

Method	Average Detection Accuracy
CMSCNN	98.06%
Baseline CNN	91.35%
LSTM	89.32%
SVM	87.96%

**Table 6:** Demagnetization severity detection accuracy under different signal-to-noise ratio conditions.

Method	Clean	SNR = 20 dB	SNR = 10 dB	SNR = 5 dB
CMSCNN	98.06%	97.53%	95.64%	92.07%
Baseline CNN	91.35%	90.34%	87.36%	84.75%

**Table 7:** Parameters of the 12-Slot, 10-Pole PMSM.

Parameter	Value	Parameter	Value
Axial length/mm	125	Stator inner diameter/mm	39
Rotor outer diameter/mm	34	Air gap length/mm	2
Slot number	12	Pole number	10
Phase number	3	Parallel branch number	2
Permanent magnet material	N48UH	Winding turns	54

**Table 8:** Motor operating condition acquisition table.

No.	Speed (r/min)	Torque (N·m)
1	500	4
2	500	6
3	800	4
4	800	6
5	1000	4
6	1000	6

**Table 9:** Demagnetization severity detection accuracy.

	Group 1	Group 2	Group 3	Group 4	Group 5	Group 6
Accuracy	92.67%	92.83%	94.52%	92.36%	93.15%	94.53%

## Data Availability

The original contributions presented in the study are included in the article. Further inquiries can be directed to the corresponding authors.
